# Novel method for rapid toxicity screening of magnetic nanoparticles

**DOI:** 10.1038/s41598-018-25852-4

**Published:** 2018-05-10

**Authors:** A. Erofeev, P. Gorelkin, A. Garanina, A. Alova, M. Efremova, N. Vorobyeva, C. Edwards, Y. Korchev, A. Majouga

**Affiliations:** 1 National University of Science and Technology «MISIS», Leninskiy prospect 4, 119049 Moscow, Russian Federation; 2 Lomonosov Moscow State University, Leninskiye gory, GSP-1, 119991 Moscow, Russian Federation; 3 Medical Nanotechnology LLC, Skolkovo Innovation Center, 121205 Moscow, Russian Federation; 4 National Research Center «Kurchatov Institute», Akademika Kurchatova pl. 1, 123182 Moscow, Russia; 5 Department of Medicine, Imperial College London, London, W12 0NN United Kingdom; 6 Dmitry Mendeleev University of Chemical Technology of Russia, Miusskaya sq. 9, 125047 Moscow, Russia; 7 WPI Nano Life Science Institute (WPI-NanoLSI), Kanazawa University, Kakuma-machi, Kanazawa 920-1192 Japan

## Abstract

Iron oxide nanoparticles have attracted a great deal of research interest and have been widely used in bioscience and clinical research including as contrast agents for magnetic resonance imaging, hyperthermia and magnetic field assisted radionuclide therapy. It is therefore important to develop methods, which can provide high-throughput screening of biological responses that can predict toxicity. The use of nanoelectrodes for single cell analysis can play a vital role in this process by providing relatively fast, comprehensive, and cost-effective assessment of cellular responses. We have developed a new method for *in vitro* study of the toxicity of magnetic nanoparticles (NP) based on the measurement of intracellular reactive oxygen species (ROS) by a novel nanoelectrode. Previous studies have suggested that ROS generation is frequently observed with NP toxicity. We have developed a stable probe for measuring intracellular ROS using platinized carbon nanoelectrodes with a cavity on the tip integrated into a micromanipulator on an upright microscope. Our results show a significant difference for intracellular levels of ROS measured in HEK293 and LNCaP cancer cells before and after exposure to 10 nm size iron oxide NP. These results are markedly different from ROS measured after cell incubation with the same concentration of NP using standard methods where no differences have been detected. In summary we have developed a label-free method for assessing nanoparticle toxicity using the rapid (less than 30 minutes) measurement of ROS with a novel nanoelectrode.

## Introduction

In the last decade, magnetic nanoparticles (MNPs), especially superparamagnetic iron oxide nanoparticles (SPIONs), have been extensively researched in biomedicine for their potential use in both diagnosis and therapy^1^. Among the most promising nanoparticles, SPIONs are the only magnetic nanoparticles that have been approved for clinical use to date^2^. SPIONS such as magnetite, Fe_3_O_4_ and maghemite, γ-Fe_2_O_3_ have attracted a great deal of research interest and have been widely used in bioscience and clinical research, including tissue repair^3,4^, cell sorting^5^, targeted drug delivery^6^, contrast agents for magnetic resonance imaging (MRI)^7^, hyperthermia and magnetic field assisted radionuclide therapy^8,9^. However it is clearly very important to appreciate whether the growing application of iron oxide MNPs or engineered nanoparticles can cause damage either to the environment or to the patient. Many reviews have been published suggesting that there may be a correlation between the mechanism of toxicity of iron oxide MNPs and major physicochemical factors responsible for *in vitro*/*in vivo* toxicity^10–12^.

The application of iron oxide has several risks including cytotoxicity with impairment of mitochondrial and nuclear functions^13–15^. Not surprisingly considerable effort has been made to investigate the potential adverse biological effects and safety issues associated with SPIONs. Many studies have demonstrated a range of toxic effects associated with exposure to nanomaterials, including mitochondrial damage, oxidative stress, chromosomal and oxidative DNA damage, altered cell cycle regulation and protein denaturation^16,13,17–20^, However very little is still known about the underlying mechanisms responsible for the toxic actions of nanoparticles. Most work to date has suggested that ROS generation (which can be either protective or harmful during biological interactions) and consequent oxidative stress are frequently observed with NP toxicity^13,21^. Reactive oxygen species are key molecules released during the transmission of cellular signals and in homeostasis. The species includes ROS-superoxide anion (O2-), hydroxide radical (OH), hydrogen peroxide (H2O2), singlet oxygen (O2), hypochlorous acid (HOCl). The physicochemical characters of NP including particle size, surface charge, and chemical composition are key indicators of the resulting ROS response and NP-induced injury since many of these NP intrinsic properties can catalyze ROS production^22^. NP-mediated ROS responses have been reported to orchestrate a series of pathological events such as genotoxicity, inflammation, fibrosis, and carcinogenesis.

The chemical composition and crystalline nature of SPIONs have also been linked to ROS related redox reactions. The Fenton-like reaction was significantly affected in terms of increased H_2_O_2_ production by the higher ratios of iron (II, III) at neutral pH levels^23^. Moreover, the stoichiometric ratio of Fe^2+^ and Fe^3+ ^^24^ and oxidation states (magnetite and maghemite) respond differently toward the redox activity and production of hydroxyl radicals^25,26^.

Establishing the role of oxidative stress requires the ability to measure its mediators accurately^27^. There is thus a need to develop improved sensitive and specific methods to detect and evaluate the level of reactive oxygen species in biological samples^28^. In an attempt to do this, spectroscopic techniques such as fluorescence, electron spin resonance and chemiluminescence have been applied to monitor ROS production^29–31^.

Optical methods are currently most often used for intracellular detection of ROS. These have a number of drawbacks including extensive sample preparation, the use of labels that can influence the formation of ROS, the multistage and complexity of the techniques used, an inability to measure ROS within a single cell, and the need for highly qualified operators. Recently a novel tool has been developed to dynamically probe the molecular mechanisms responsible for the oxidative cell response^32^. Most of the optical methods are useless for measurement over long periods of time because of fast inactivation of the fluorescent dyes used and are specific only to particular ROS (mainly to H2O2^33^).

In this situation electrochemical sensor systems are a logical choice for ROS detection because of their high portability and cost effectiveness, and applicability for real time *in vitro* and *in vivo* measurements^34–36^. A new macro amperometric biosensor for hydrogen peroxide and superoxide anion has been developed. The biosensor developed uses Cytochrome C modified glassy carbon electrodes coupled with electrochemically reduced graphene oxide^37^. However it is not applicable for single cell live measurements due to its size and its sensitivity is inadequate for intracellular analysis. Early nanopipettes have proved to be powerful instruments for single cell analysis, including high resolution topographical imaging of living cells^38^, quantitative delivery of molecules to the surface of living cells^39^, nanoscale targeted patch clamp measurements in neuronal cultures^40^, and hold great promise as intracellular biosensors^41^. Mirkin’s group pioneered the application of electrochemical probes for the measurement of redox properties of living cells^42^. A joint effort between Amatore and Mirkin’s groups demonstrated the application of platinized nanoelectrodes for the intracellular detection of ROS species inside murine macrophages^43^. However, despite the nanometer dimension of the electro-active area, the outer glass coating was several hundred nanometers. Recently Zhang X.W. and colleagues showed quantitative measurement of ROS/RNS in individual phagolysosomes of living macrophages with cylindrical nanowires electrodes^44^. The size of the platinized nanowire electrode was 200–500 nm.

We have previously reported the fabrication of disk-shaped carbon nanoelectrodes based on a quartz nanopipette whose radius could be precisely tuned within the range 5–200 nm. The functionalization of the nanoelectrode with platinum allowed electrochemical ROS measurements within melanoma cells with minimal disruption of cell function^45^. We described how such disk-shaped platinized carbon electrodes could be very easily produced and that their diameter was comparable to that of a platinized nanowire electrode^44^. For the rapid screening of magnetic nanoparticles toxicity it is necessary to have electrodes with very stable catalytic activity. In^45^ we reported a carbon disk-shaped nanoelectrode with platinum deposited on its top. Its drawback was that platinum could be removed during penetration into the cell. Here we report a new type of nanoelectrode based on a quartz nanopipette with better adhesion of platinum. We demonstrate the use of this for the rapid screening of iron oxide magnetic nanoparticle toxicity.

## Results and Discussion

### Production of electrodes

Commercially available disk shaped carbon nanoelectrodes isolated in quartz (ICAPPIC Limited, UK) with diameters 60–100 nm were used in all experiments. Carbon itself is a relatively inert material and to detect specific redox-active species further functionalization is needed. An electrodeposited platinum layer enhances the electrocatalytic activity by drastically reducing the overpotential produced by the reduction of oxygen and the oxidation/reduction of hydrogen peroxide. Platinization of such carbon electrodes has been used for intracellular ROS measurements in melanoma cancer cells^45^. These platinized electrodes have low adhesion of platinum and are not suitable for multiple toxicity experiments. In this paper a nanocavity etched into the carbon electrode was used to enhance the adhesion of the platinum to the carbon plug.

**Figure 1:**
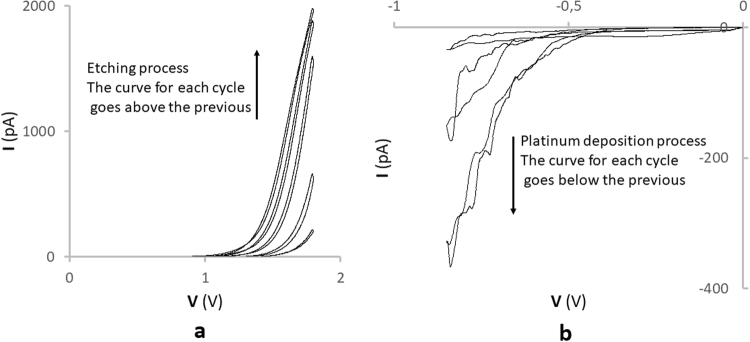
(**a**) Shows current-voltage characteristic recorded during the process of etching the carbon nanoelectrode in a 0.1 M NaOH, 10 mM KCl solution during 40 cycles for 10 seconds each to create a cavity on the nanoelectrode surface. The curve for each cycle goes above the previous. (**b**) Shows current-voltage characteristic recorded during the platinum deposition process carried out by sweeping the potential from 0 to −800 mV vs Ag/AgCl in a solution containing 2 mM PtCl.

We have used an electrochemical method to create cavities in carbon nanoelectrodes^46^. Fabrication of platinum nanoelectrodes was performed in two stages: etching in alkaline solution and platinization. All stages were precisely controlled by electrochemical measurements (Fig. 1). We etched the carbon nanoelectrode in a 0.1 M NaOH, 10 mM KCl solution during 40 cycles of 10 seconds each to create a cavity on the nanoelectrode surface (Fig. 1a). Platinum deposition was carried out by sweeping the potential from 0 to −800mV vs Ag/AgCl in a solution containing 2 mM PtCl. We obtained cyclic voltammograms of the electrode in 1 mM ferrocene methanol (FcMe) for the initial carbon electrode, for the electrode with nanocavities and with platinum (Fig. 1b).

**Figure 2:**
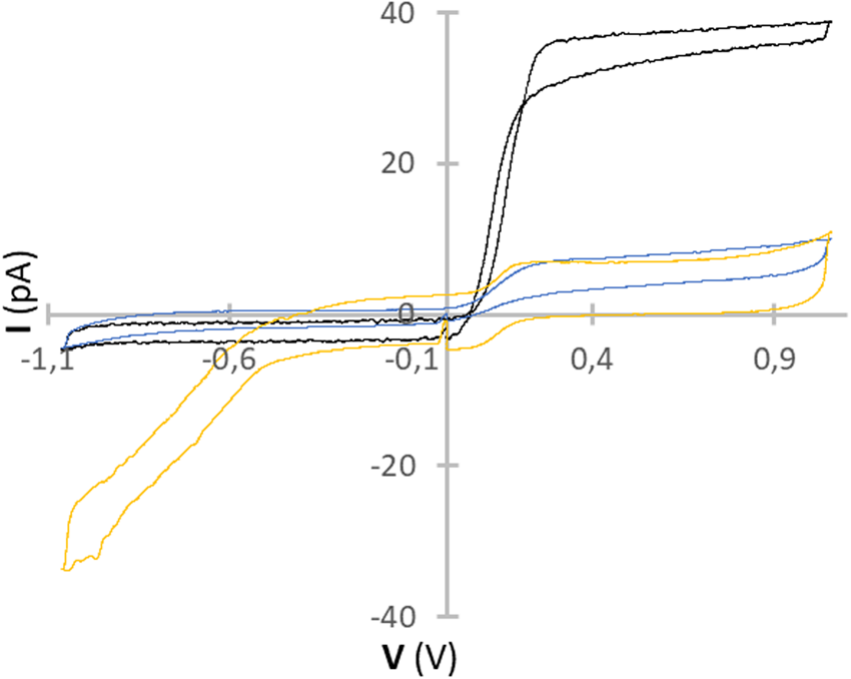
Electrochemical characterization of nanoelectrodes vs Ag/AgCl. Cyclic voltammograms in 1 mM ferrocene methanol in PBS of a nanoelectrode: black graph – disk shape initial carbon electrode; blue graph – electrode after etching in NaOH solution; yellow graph – platinized electrode.

According to cyclic voltammograms (CVs) of nanoelectrode in FcMe the charge of the carbon outer surface was slightly decreased after etching (Fig. 2). It has proven challenging to image nanoelectrodes smaller than ∼100 nm by SEM, and alternative electrochemical measurements need to be used for characterization of size. We have compared our signal in FcMe with similar graphs of common carbon disk shaped nanoelectrodes^47^. As shown in Fig. 2 the size of the tip of our electrodes is less than 100 nm.

The platinized nanoelectrode showed increased catalytic activity for oxygen reduction. The deposition of Pt only slightly increased the effective geometric surface area of the nanoelectrode (as evidenced by the voltammogram for the oxidation of 1 mM FcMeOH), but dramatically enhanced its catalytic activity toward oxygen reduction (Fig. 2).

To confirm the nanosize and platinum presence on the tip of the electrode we obtained SEM images with EDX analysis. We imaged the tip and performed EDX analysis of the electrode at all stages before and after platinization. The size of the electrode did not change at any stage and platinum was detected only at the last stage after platinization [see Additional file 1: Fig. S1].

**Figure 3:**
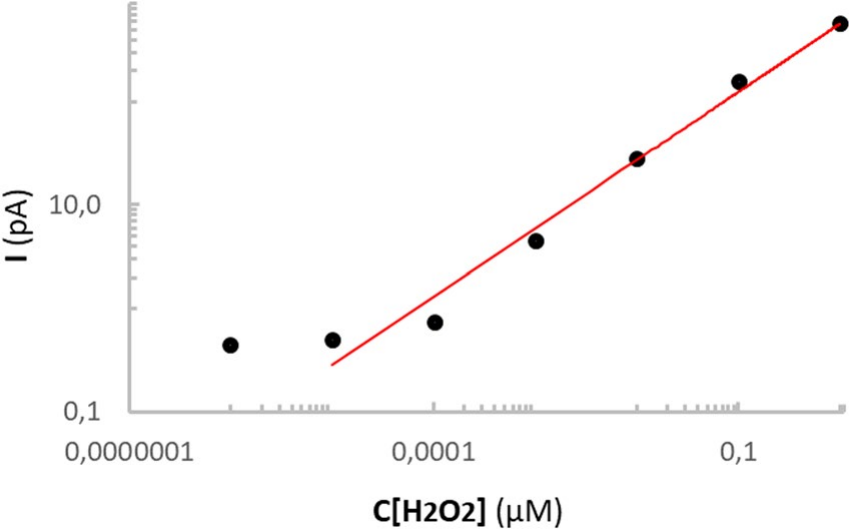
Dose response curve of the oxidation of hydrogen peroxide by the platinized nanoelectrode (at +800 mV vs Ag/AgCl).

To estimate the amount of ROS in our measurements and to show the concentration dependence of current at our platinized electrode we calibrated the anodic response with H2O2. We also carried out chronoamperometry measurements with the nanoelectrode at a potential of +800 mV in PBS bulk solution. We then added hydrogen peroxide in bulk solution (Fig. 3). The difference between 0.1 and 1 uM of H2O2 can be easily recognized. Dose dependent concentration is linear in a range from 0.1 to 10000 uM of H2O2. Our system does not have the sensitivity to distinguish concentrations below 0.1 uM.

All our electrodes were calibrated before intracellular measurements. Dose response curves for different electrodes had similar sensitivity and linearity in the measurement range [see Additional file 1: Fig. S2].

We have thus demonstrated that it is possible to fabricate sensitive platinized electrodes with enhanced adhesion of platinum, hidden inside etched cavities. All stages can be easily controlled by electrochemical measurement. Each electrode can be used for several penetrations of cell membranes and ROS measurements before and after incubation with iron oxide NPs. It must be noted that the platinized electrode can measure all type of ROS such as superoxide radical, hydroxyl radical and peroxide at +800 mV vs Ag/AgCl^43^.

### Iron oxide NP toxicity

It has previously been reported that cell incubation with iron oxide NPs results in intracellular ROS production^48,49^. We have used a 10 nm diameter iron oxide NP as a model to test NP toxicity (Magn)^50^. Pluronic F127 was chosen for the purpose of stabilization since it has already been shown to increase the biocompatibility of Magn NPs, preventing aggregation, protein adsorption and ROS recognition^51^. We thought that Magn covered with Pluronic F127 (Magn-Plu) would represent a less toxic model of NP which should produce lower level of ROS. For the synthesis of Magn and Magn-Plu NPs we used a co-precipitation method, which is both simple and versatile, allowing the addition of polymer stabilizers during the process of NP formation. The size, shape and composition of Magn and Magn-Plu were confirmed by XRD-analysis, DLS, TEM methods [see Additional file 1: Figs S3–S5].

### Screening of magnetic nanoparticle toxicity on living cells

**Figure 4:**
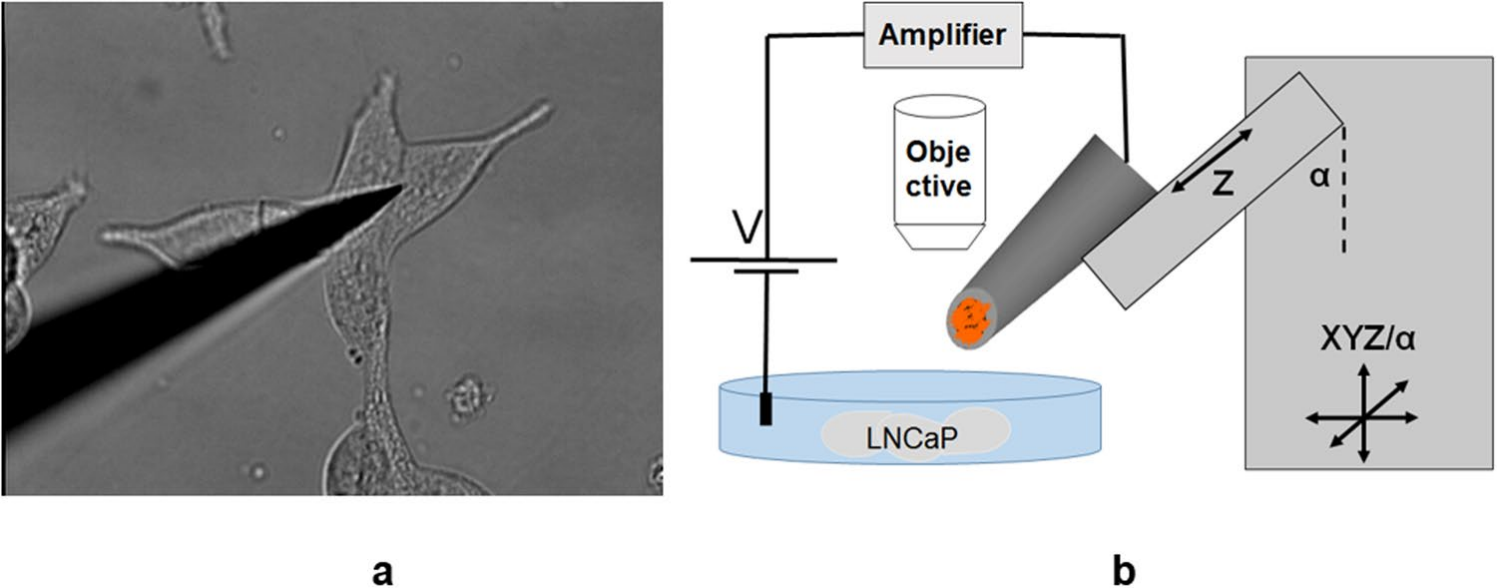
(**a**) Photomicrograph of intracellular measurement of ROS in LNCaP cells by nanoelectrode. (**b**) Schematic diagram showing the intracellular measurement system, key components and the movements in X, Y, and Z directions that could be achieved.

We carried out a comparative study of the cytotoxicity of Magn and Magn-Plu NP by intracellular ROS measurements with platinized nanoelectrodes with enhanced cavity-based adhesion. We performed intracellular ROS measurements in HEK293 and LNCaP cancer cells before and after exposure to 10 nm size iron oxide NP. Intracellular measurements were carried out using a laboratory setup based on the PatchStar micromanipulator (Fig. 4a,b). The approach angle of the micromanipulator can be adjusted between 0 and 90 degrees to the horizontal plane, which allows penetration into cells under high magnification objectives that have a short working distance. The platinized nanoelectrode can be precisely inserted into an individual cell on the Petri dish to monitor intracellular molecules.

**Figure 5:**
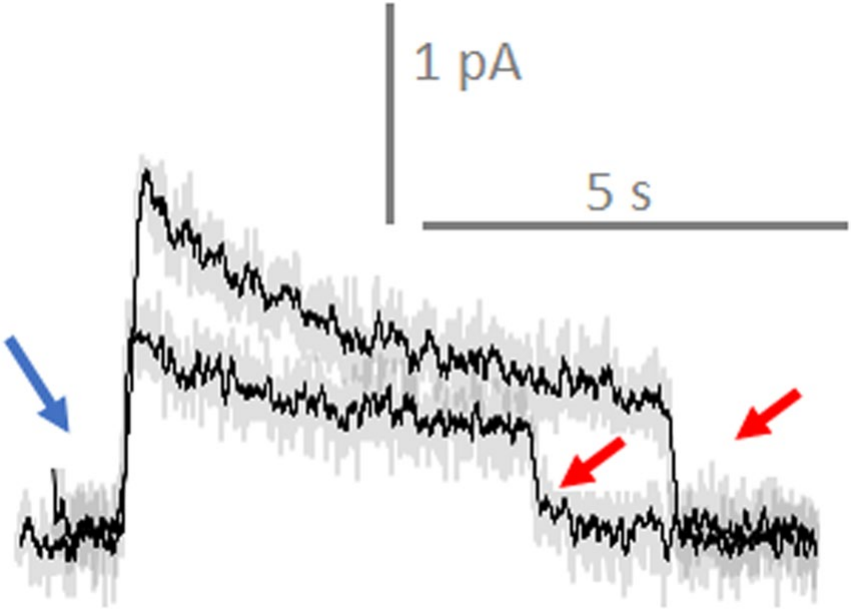
ROS intracellular measurements in individual HEK293 cells. Voltammograms before and after penetration of cells with the nanoelectrode. Voltage is applied vs Ag/AgCl. Representative current traces of a nanoelectrode polarized at +800 vs Ag/AgCl inside and outside HEK293 cells. Red and blue arrows indicate, respectively, the moment of penetration and retraction. Top graph - ROS intracellular measurements in cells incubated with Magn NP for 3 h in HBSS, lower graph - ROS intracellular measurements in pure cells after 3 h in HBSS. Grey curves are pure records, black curves are records with 100 averaging.

**Figure 6:**
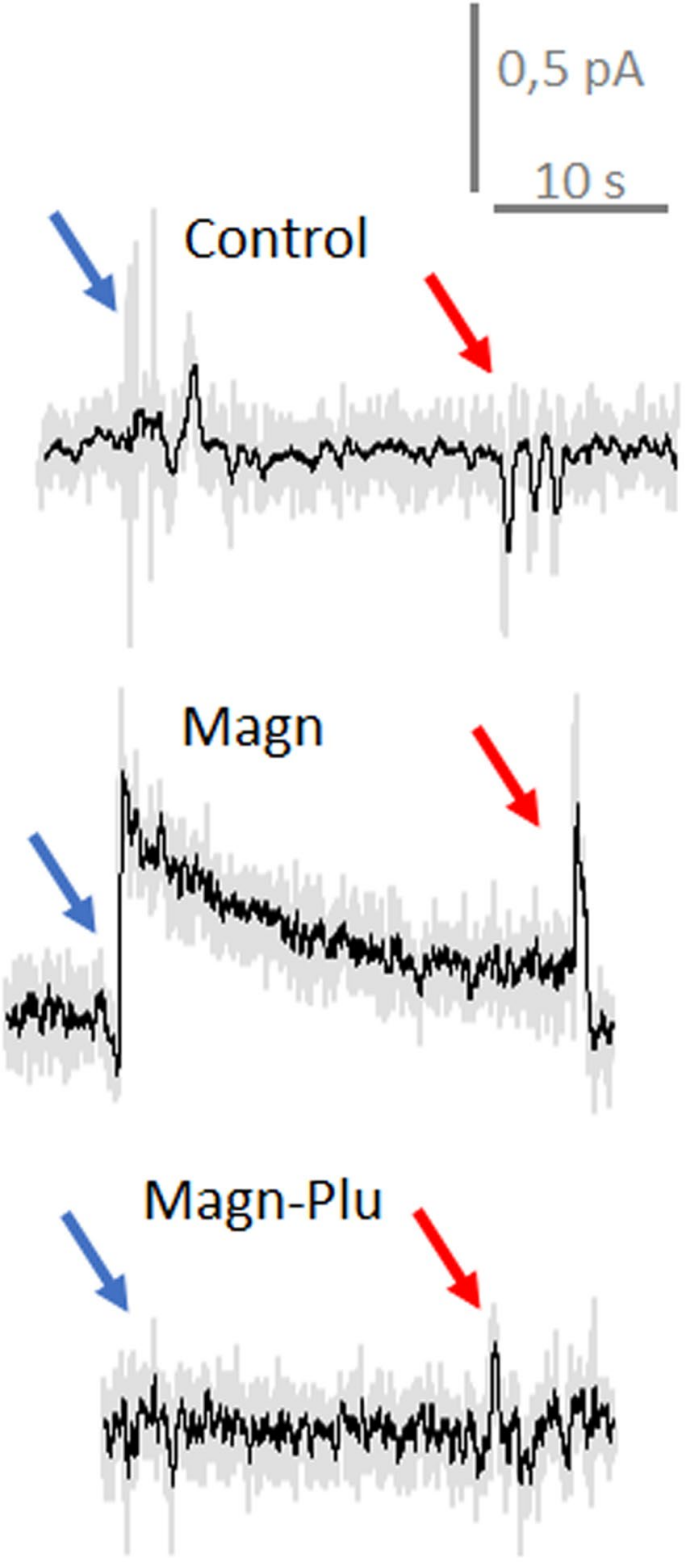
ROS intracellular measurements in individual LNCaP cells. Voltammograms before and after penetration of cells with the nanoelectrode. Voltage is applied vs Ag/AgCl. Representative current traces of a nanoelectrode polarized at +800 vs Ag/AgCl inside and outside LNCaP cells. Red and blue arrows indicated, respectively, the moment of penetration and retraction. Similar traces were obtained from 5 different cells from each sample by the same electrode. Control – ROS intracellular measurements in pure cells after 3 h in HBSS; Magn - ROS intracellular measurements in cells incubated with Magn NP during 3 h, Magn-Plu - ROS intracellular measurements in cells incubated with Magn-Plu NP during 3 h. Grey curves are pure records, black curves are records with 100 averaging.

During the penetration of individual HEK293 and LNCaP cells (Figs 5 and 6) we consistently elicited similar chronoamperometry measurements (with the nanoelectrode poised at a potential of 800 mV vs Ag/AgCl). Values of currents during single cell metabolite measurements are usually very small and recording of Faradaic current induced by ROS from one cell is challenging^45,52^. The electrochemical signal was measured with a patch clamp amplifier filtered using a low-pass filter at 1000 Hz. In experiments we used Axon Digidata 1322 A digitized at an aggregate speed of 500 kHz. During penetration we postulated that there might be transient effects and thus tried to do less filtering and averaging during the recording. In Fig. 6 the curves are presented with and without 100 point averaging. The anodic current quickly increased followed by relatively slow equilibration, and then a rapid return to the baseline after retraction of the nanoprobe from the cell (Figs 5 and 6). Red and blue arrows indicate the respective moment of penetration and retraction. The penetration of 5 different cells with the same nanoelectrode generated a reproducible intracellular anodic current. We always checked catalytic activity of the platinized electrode during intracellular measurements by comparing the shape of the cyclic voltammograms in HBSS buffer solution between measurements [see Additional file 1: Fig. S6].

We carried out comparative measurements before and after exposure of HEK293 cells to Magn (at a concentration 8.56 μg/ml) for 3 h in HBSS buffer solution. The presence of Magn NP dramatically increased ROS generation inside the cell (Fig. 5). As shown in Fig. 3 we could then estimate the level of intracellular ROS (0.2 uM H2O2 before and 0.5 uM H2O2 after exposure with Magn NP).

We carried out similar measurements with LNCaP cells which do not produce detectable level of ROS without Magn NPs (Fig. 6 – Control). Presence of Magn NPs markedly increased ROS generation inside cells (Fig. 6 – Magn). Covering the Magn NP with biocompatible Pluronic F127 decreased the intracellular level of ROS to that seen initially in HBSS. Magn-Plu NP did not induce a detectable rise of intracellular ROS inside LNCap cells after 3 h exposure to a concentration of 8.56 μg/ml. As shown in Fig. 3 we could estimate the level of intracellular ROS as 0.1 uM H2O2 after exposure of LNCaP cells to Magn NP. All measurement took less than 30 min to test the ROS level in a single cell line with one electrode.

**Figure 7:**
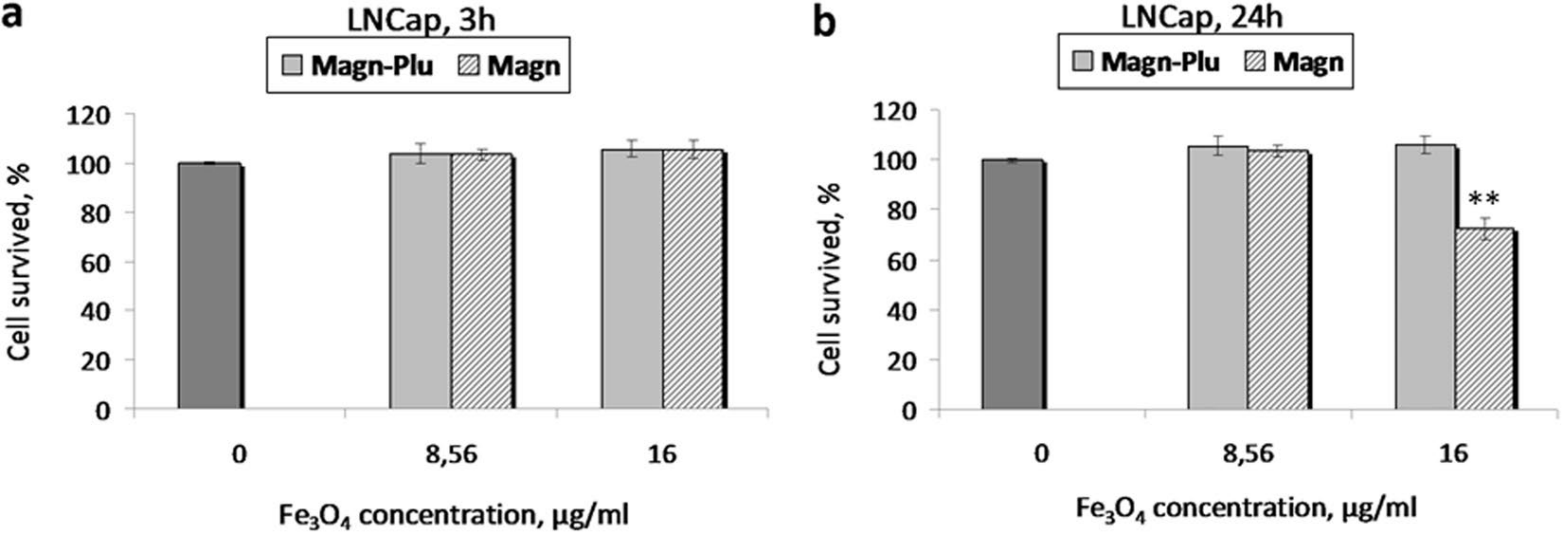
Histograms of LNCaP cell viability after incubation with two types of superparamagnetic iron oxide NPs for 3 h (**a**) and 24 h (**b**). MTS assay. Results are shown as means ± SD. **P < 0.01 (one-way ANOVA). comparing to control cells.

We carried out a comparative study of the cytotoxicity of Magn and Magn-Plu NP using well established methods. The effect of NPs on living cells was analyzed by standard MTS assay. The data demonstrated that incubation of cells with Magn and Magn-Plu NPs for 3 h did not result in a decrease in cell viability (Fig. 7a). A substantial difference in the cytotoxic effects of Magn-Plu and Magn NPs was revealed after 24 h of their exposure to cells. Magn NPs caused 30% cell death (P < 0.01) (Fig. 7b).

**Figure 8:**
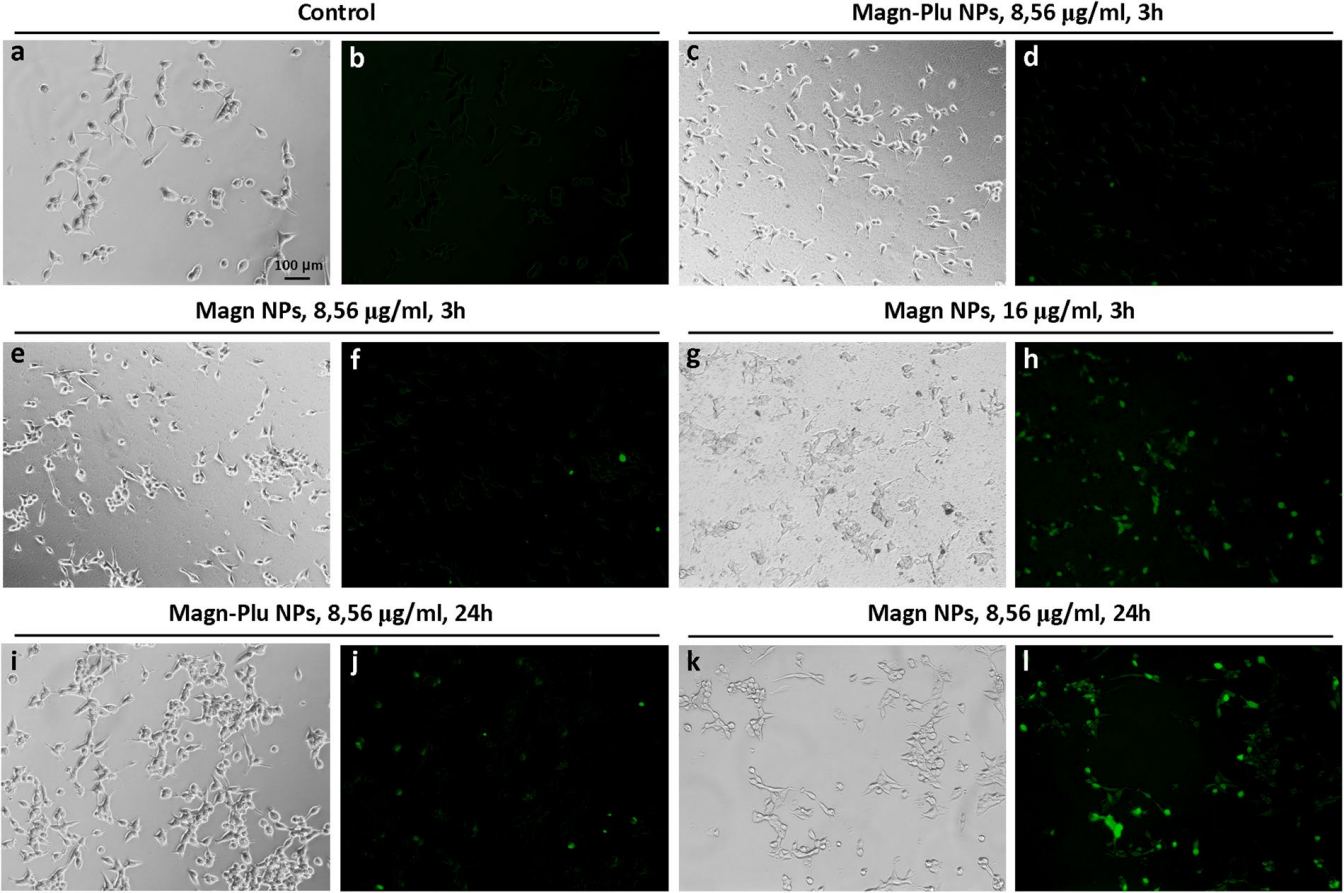
Intravital H2DCFDA staining of LNCaP cells incubated with Magn-Plu and Magn NPs during 3 and 24 h. (**a,c,e,g,i,k**) – phase contrast microscopy; (**b,d,f,h,j,l**) – fluorescent microscopy.

A more sensitive standard method for cytotoxicity study is ROS detection by cell staining with 2′,7′-dichlorodihydrofluorescein diacetate (H2DCFDA). This was used to evaluate the NP effect on cell viability. Analysis of samples obtained during LNCaP cell incubation with 8.56 μg/ml Magn and Magn-Plu NPs for 3 h as well as control cell samples,cultivated without NPs, revealed single H2DCFDA-positive cells (Fig. 8a–f). Based on their morphology we assume that these cells underwent apoptosis: they were round-shaped, small in size and their cytoplasm dense^53^. H2DCFDA fluorescent diffuse staining of flattened cells was detected only in cells incubated with Magn NPs at a concentration of 16 μg/ml (Fig. 8g,h). Magn-Plu NPs exposed to cells for 24 h at concentrations 8.56 and 16 μg/ml resulted in an increase of the number of round-shaped cells stained with H2DCFDA (Fig. 8i,j). However, this increase was insignificant. At the same time, incubation of cells with Magn NPs caused marked amplification of ROS generation (Fig. 8k,l).

**Figure 9:**
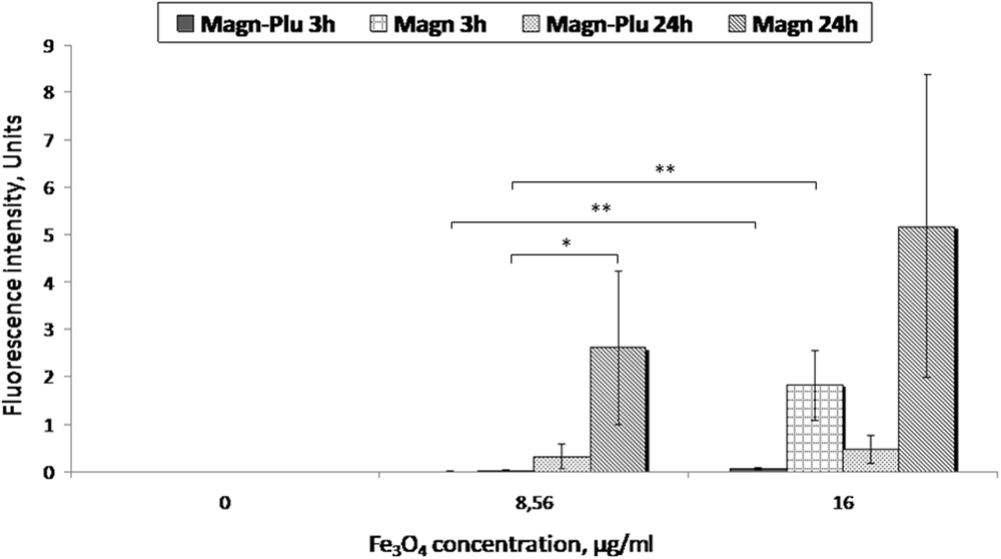
Histogram of H2DCFDA fluorescence intensity in LNCaP cells after incubation with Magn-Plu and Magn NPs for 3 h and 24 h. Results are shown as means ± SD. **P < 0.01, *P < 0.05 (one-way ANOVA).

Based on the obtained images we calculated histograms of H2DCFDA dye fluorescence intensity in LNCaP cells after incubation with Magn-Plu and Magn NPs at different concentrations and times. The level of ROS in control cells was taken as zero. These results demonstrate that ROS content in cells incubated with Magn and Magn-Plu NPs at a concentration of 8.56 μg/ml for 3 h is not detectable by this fluorescent method. Signals increased significantly at a NP concentration of 16 μg/ml (P < 0.01) (Fig. 9). The level of ROS in cells cultured with Magn-Plu and Magn NPs for 24 h was on average higher than after 3 h of incubation. It must be noted that all methods showed that all concentrations of Magn NPs caused more active production of ROS than Magn-Plu NPs when incubated with LNCaP cells.

**Figure 10:**
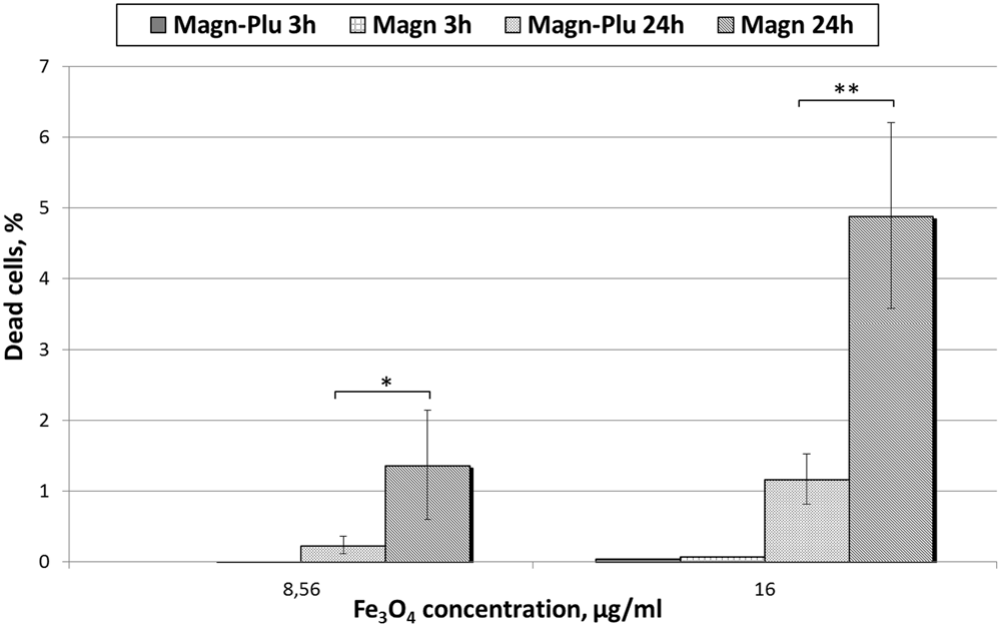
Histogram of apoptotic cells percentage in LNCaP cells population after incubation with Magn-Plu and Magn NPs for 3 h and 24 h. Results are shown as means ± SD.

Finally, standard intravital fluorescent staining of cells with an apoptosis/necrosis kit was prepared. The results demonstrated that both concentration (8.56 and 16 μg/ml) of Magn and Magn-Plu NPs did not increase the percentage of dead cells after 3 h co-incubation with cells in comparison with samples cultivated with water (Fig. 10). Only after 24 h of exposure to NPs was there a significant increase in the number of dead cells in the population (Fig. S7). It must be noted that Magn NPs were more toxic than Magn-Plu NPs.

While excess levels of ROS are known to induce apoptosis^54,55^, we can conclude on the basis of the results obtained from the MTS assay and H2DCFDA and apoptosis/necrosis staining that only Magn NPs at a concentration of 16 μg/ml incubated with cells for 24 h caused an increase in ROS production that was sufficient to induce cell death. However, a lower increase in the levels of ROS can lead to intracellular organelle and DNA damage, with consequent apoptosis. Thus, it is likely to be useful to be able to detect even very small increases of ROS levels inside the cell to help predict the cytotoxic effect of NPs. The MTS assay had a very low sensitivity and did not demonstrate a detectable signal even at high concentration after 3 h incubation. Fluorescent assays showed better sensitivity then MTS testing but did not achieve the detection limit found in our novel screening method using electrochemical ROS sensing after 3 h of incubation. It must be noted that the fluorescent method required an additional preparation stage for incubation of cells with H2DCFDA. This makes cytotoxicity studies both longer and more complicated.

## Conclusions

We have developed a novel tool for single cell ROS measurements and have shown that this has the potential to be used to study the toxicity of iron oxide based nanoparticles. The method is simple and the cost effective method of platinized electrochemical nanoprobe fabrication makes the technology very promising for biomedical application. We have shown how to produce sensitive platinized electrodes with enhanced adhesion of platinum that can be used for screening of intracellular ROS. We conclude that our label free novel method for rapid screening of magnetic nanoparticles toxicity is more sensitive and faster than the standard commercially available methods for studying NP cytotoxicity.

## Methods

### Cell Culture

LNCaP (ATCC CRL-1740) human prostate adenocarcinoma cell line was cultured in RPMI-1640 medium (gibco) supplemented with 10% Fetal Bovine Serum (Sigma), 2 mM L-glutamine (gibco), antibiotic (0,1 Units/ml penicillin, 0,1 µg/ml streptomycin) (gibco) and RPMI vitamin solution (Sigma). HEK293 (ATCC CRL-11268G-1) human embryonic kidney cell line was cultured in DMEM medium (gibco) supplemented with 10% Fetal Bovine Serum (Sigma), 2 mM L-glutamine (gibco) and antibiotic (0,1 Units/ml penicillin, 0,1 µg/ml streptomycin) (gibco). Cells were maintained at 37 °C in a humidified incubator supplied with 5% CO2.

### Materials

Iron chloride (II) tetrahydrate, iron (III) chloride, Pluronic F127, ammonium hydroxide solution, (28.0–30.0%), hydrochloric acid (36%), iron standard for ICP (TraceCERT, 1000 mg/L Fe in nitric acid) were purchased from Sigma-Aldrich. All water used in experiments was deionized (18.2 MΩcm^−1^, Millipore Milli-Q Academic System). All vessels were washed with hot solution of aqua regia and then rinsed with DI water before making syntheses.

### Synthesis of Fe3O4 Nanoparticles (NPs)

Fe3O4 NPs with/without Pluronic F127 coating were prepared by co-precipitation of Fe(II) and Fe(III) salts (Supporting information).

### MTS assay

Cells were plated at concentration of 10 000 cells per well in 96-well plates. The solutions of Pluronic F127 stabilized and uncovered MNPs in distilled water were added to the cells after one day (the final concentrations of magnetite are shown at the histogram (Fig. 7)). Distilled water (20%) and DMSO (25%) were taken as negative and positive controls, correspondingly. Cells were incubated with these MNPs for 3 and 24 h. Then cells were washed with PBS, and 20 μl of MTS reagent (CellTiter 96 AQueous Non-Radioactive Cell Proliferation Assay, Promega, USA) was added to each well with 100 μl of culture medium. After 4 h incubation at 37 °C in darkness, the absorbance of the solution was measured at 490 nm using Thermo Scientific Multiskan GO spectrometer.

### ROS detection by 2′,7′-dichlorodihydrofluorescein diacetate (H2DCFDA)

Cells were seeded on glass coverslips at a concentration 100 000 cells per ml in 24-well plates and cultured at 37 °C in a humidified incubator supplied with 5% CO2. After a day both samples were added to cells for 3 and 24 h. Following final concentrations were analyzed: for Magn and Magn-Plu 8,56 µg/ml and 16 µg/ml. To detect ROS in cells after MNPs addition, unfixed cells were washed with HBSS supplemented with 2 mM L-glutamine and 10 mM HEPES (pH 7,4 adjusted with 1 N NaOH), and stained with 2 µM H2DCFDA solution (life technologies) for 30 min at 37 °C in darkness. Then cells were carefully washed with HBSS 4 times for 5 min. The obtained preparations were analyzed at fluorescence microscope EVOS (life technologies), objective PlanFluor 20x/0.45. The further processing of the photos was carried out by ImageJ software as well as fluorescence intensity calculation (40–60 cells/time point for each concentration).

### Apoptosis/necrosis detection

Cells were plated in 96-well plates at concentration of 10 000 cells per well. After one day the solutions of Magn-Plu and Magn NPs in distilled water were added to the cells in final concentrations 8,56 µg/ml and 16 µg/ml for 3 and 24 h. Cells incubated with distilled water were used as control. Cells were twice washed with HBSS supplemented with 2 mM L-glutamine and 10 mM HEPES, intravital stained with Apoptosis/Necrosis detection kit (abcam) for 40 min at room temperature, and washed with full HBSS 2 times again. The obtained preparations were analyzed at fluorescence microscope EVOS (life technologies), objective PlanFluor 20x/0.45. The further processing of the photos was carried out by ImageJ software. The number of apoptosis/necrosis positive cells was counted per at least 1,000 cells and expressed as percent. Percentages of dead cells in the control samples were taken as zero.

### Statistical analysis

The frequency of dead cells in the experiment with apoptosis/necrosis kit staining was represented as a percentage (mean ± SD) of the total cells counted (n = 1,000 cells). All data were obtained in three independent triplicate experiments. Plotting and calculation of the standard deviation value were made using Microsoft Office Excel 2007 software. P value was calculated using One-way ANOVA calculator. P values < 0.05 were considered significant.

### Electrochemical measurements

The nanoelectrode was back contacted with a silver wire and the second Ag/AgCl electrode was placed in a bulk solution acting as an auxiliary/reference electrode. All potentials are quoted against this electrode. The Faradaic current was measured with a MultiClamp700B patch clamp amplifier (Axon Instruments). The electrochemical signal was filtered using a low-pass filter at 1000 Hz and digitized with an Axon Digidata 1322 A (Axon Instruments), and a PC equipped with pClamp 10 software (Molecular Devices).

### Cyclic voltammetry

Cyclic voltammetry was measured in a solution of 10 mM hexaammineruthenium(III) chloride (Sigma-Aldrich) or 1 mM ferrocene methanol (Sigma-Aldrich) in PBS (phosphate buffered saline solution having pH of 7.4 and prepared from 7.2 mM Na_2_HPO_4_, 2.8 mM KH_2_PO_4_, and 150 mM NaCl). Etching the carbon nanoelectrode was carried out in a 0.1 M NaOH, 10 mM KCl solution during 40 cycles for 10 seconds each to create a cavity on the nanoelectrode surface.

Carbon nanoelectrodes are platinized in a solution of chloroplatinic acid H2PtCl6 (2 mM) in 0.1 hydrochloric acid. The reduction of Pt at the carbon nanoelectrode was induced via cyclic voltammetry from 0 to +800 mV with a scan rate of 200 mV/s.

### Intracellular measurements

Cells were seeded in Petri dishes at a concentration 100 000 cells per ml and cultured at 37 °C in a humidified incubator supplied with 5% CO2. After 24 h samples of NP were added to cells for 3 h at concentration 8,56 µg/ml. Then cells were washed with HBSS 3 times. Intracellular measurements were carried out with laboratory setup based on the PatchStar micromanipulator (Scientifica, Uckfield, UK). The micromanipulator provided a coarse approach and positioning for the nanoelectrode over a 20 mm range in X, Y, and Z directions that covered most of the 35 mm diameter Petri dish sample area and also allowed the complete withdrawal of the nanoelectrode, which is necessary for sample change. The approach angle of the micromanipulator can be adjusted between 0 and 90 to the horizontal plane, which allows penetration in cells under high magnification objectives that have a short working distance. The Olympus water immersion objective LUMPlanFL N 40x, 0.8 numerical aperture, 3.3 mm working distance was used in intracellular experiments. The PatchStar micromanipulator and the sample holder were mounted on the Motorized Movable Top Plate (Scientifica, UK) that provided the coarse positioning required for the selection of the area of interest.

## Electronic supplementary material

Dataset 1
