# Supplementary material for: Novel method for rapid toxicity screening of magnetic nanoparticles
Source: Sci Rep. 2018 May 10;8:7462. doi: 10.1038/s41598-018-25852-4 (PMC5945642; doi:10.1038/s41598-018-25852-4)
Supplement: Supplementary file 1 — Dataset 1 [file 41598_2018_25852_MOESM1_ESM.docx]

**Supporting information**

**Novel method for rapid toxicity screening of magnetic nanoparticles**

Erofeev A^1,2,*^, Gorelkin P^3^, Garanina A^1,2^, Alova A^2^, Efremova M^1^, Vorobyeva N^1,4^, Edwards C^5^, Korchev Y^5^, Majouga A^1,2,6^

^1^ National University of Science and Technology «MISIS», Leninskiy prospect 4, 119991 Moscow, Russian Federation

^2^ Department of Chemistry, Lomonosov Moscow State University, Leninskiye gory 1-3, GSP-1, 119991 Moscow, Russian Federation

^3^ Medical Nanotechnology LLC, Stroiteley 4-5-47, 119311 Moscow, Russian Federation

^4^National Research Center «Kurchatov Institute», Akademika Kurchatova pl. 1, 123182, Moscow, Russia

^5^Department of Medicine, Imperial College London, London W12 0NN, United Kingdom

^6^ Dmitry Mendeleev University of Chemical Technology of Russia, Miusskaya sq. 9, 125047 Moscow, Russia

*SEM images with EDX analysis*

We imaged tip and performed EDX analysis of the electrode at all stages before and after platinization.

*
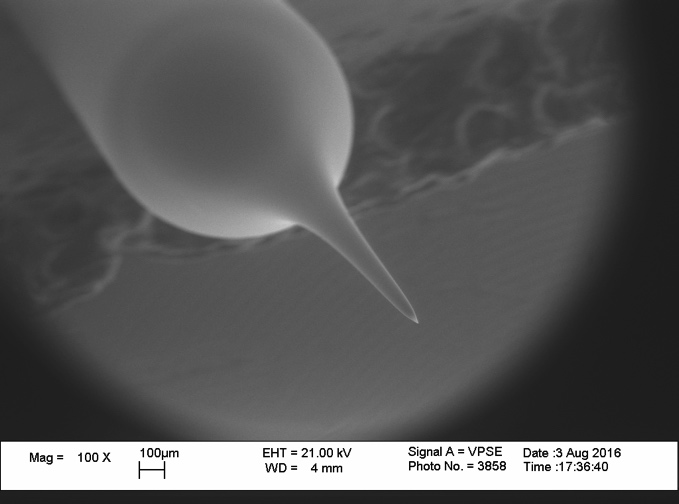
*  *
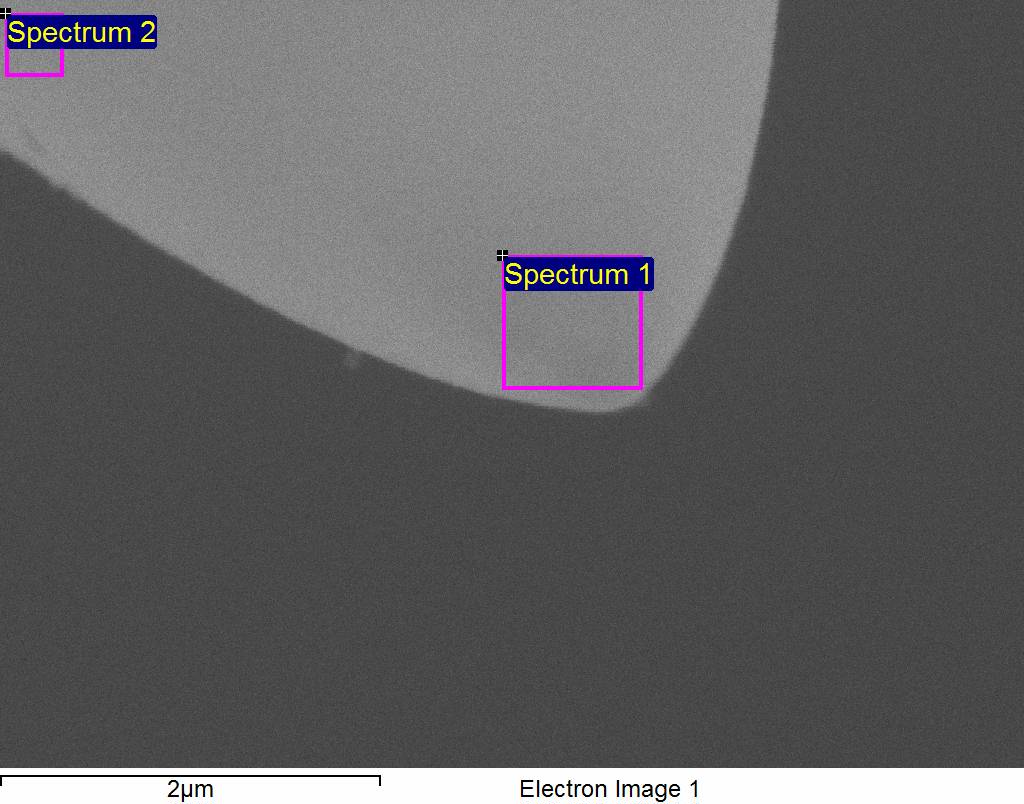
*

a b

| Spectrum | C | Al | Si | Pt | O | Total |
| --- | --- | --- | --- | --- | --- | --- |
| Spectrum 1 | 24.90 | 3.65 | 0.58 | 0.53 | 70.34 | 100.00 |
| Spectrum 2 | 24.39 | 3.98 | 1.46 |  | 70.17 | 100.00 |

c

**Fig. S1** SEM images of platinized electrode is presented (a - 100x, b – 50000x). In figure b shown zones of EDX analysis. Platinum was detected only in zone 1 on the tip of electrode. Element analysis presented in table c

*Typical dose response curves of the oxidation of hydrogen peroxide by the platinized nanoelectrodes*

*
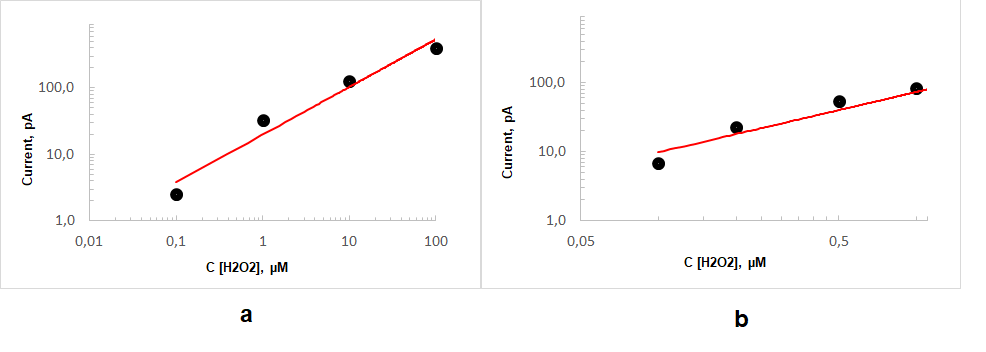
*

**Fig. S2** Dose response curves of the oxidation of hydrogen peroxide by the different platinized nanoelectrodes (at + 800 mV vs Ag/AgCl): a – for one presented wide range of concentration, b – for another one presented mainly main region of interest of concentration

*Synthesis of Fe_3_O_4_ Nanoparticles*

*Materials*

Iron chloride (II) tetrahydrate, iron (III) chloride, Pluronic F127, ammonium hydroxide solution, (28.0-30.0%), hydrochloric acid (36%), iron standard for ICP (TraceCERT, 1000 mg/L Fe in nitric acid) were purchased from Sigma-Aldrich. All water used in experiments was deionized (18.2 MΩcm^-1^, Millipore Milli-Q Academic System). All vessels were washed with hot solution of aqua regia and then rinsed with DI water before making syntheses.

*Synthesis of Magn sample*: Fe_3_O_4_ NPs without coating were prepared by co-precipitation of Fe(II) and Fe(III) salts according to the procedure described in [Lo C.K., Xiao D., Choi M.M.F. Homocysteine-protected gold-coated magnetic nanoparticles: synthesis and characterisation J. Mater. Chem. 17 (2007) 2418 – 2427] with variations. Briefly, 650 mg of FeCl_3_ and 398 mg of FeCl_2_∙4H_2_O were dissolved in 5 ml of 2М HCl, obtained solution was added dropwise to 50 ml of 0,7М NH_3_∙H_2_O solution and then the mixture was magnetically stirred for 30 minutes. After that magnetite nanoparticles were magnetically decanted and washed with 50 ml of DI H_2_O, then nanoparticles were magnetically decanted again and dissolved in 50 ml DI H_2_O for 30 minutes via ultrasonication.

*Synthesis of Magn-Plu sample*: Fe_3_O_4_ NPs with Pluronic F127 coating were prepared according to protocol described in [Hritcu D., Popa M.I., Popa N., Badescu V., Balan V. Preparation and characterization of magnetic chitosan nanospheres// Turk J Chem 33 (2009), 785 – 796] at room temperature.

*Characterization*

Transmission Electron Microscopy (TEM) measurements. TEM micrographs were obtained using JEOL JEM-1400 (100 kV) microscope. Sample was prepared by casting and evaporating a droplet of water solution of NPs onto a holey carbon-coated copper grid (300 mesh). The average diameter of NPs was calculated from TEM images by analysing of 500 NPs for each sample using Image J software (National Institutes of Health, USA).

*X-Ray Diffraction (XRD) measurements*. XRD patterns were obtained using an X-ray power diffractometer DRON-4 with CoKα radiation. The data were collected from 2*θ* = 30 to 120° at a scan rate 0,1° per step and 3 s per point. Qualitative phase analysis was performed by comparison of obtained spectra with PHAN database.

*Dynamic Light Scattering (DLS) measurements.* The hydrodynamic size and zeta potential of the magnetic NPs were measured using Nano ZS ZetaSizer (Malvern Instruments). The average particle sizes with error ranges were obtained from three measurements of each sample.

*Synthesis, size and structure of NPs*

For the synthesis of Magn and Magn-Plu NPs we used a co-precipitation method, which is both simple and versatile, allowing the addition of polymer stabilizers during the process of NPs formation. Pluronic F127 was chosen for the purpose of stabilization since it has already been shown to increase the biocompatibility of NPs, preventing an aggregation, a protein adsorption and RES recognition [E.V. Batrakova, A.V. Kabanov, Pluronic block copolymers: evolution of drug delivery concept from inert nanocarriers to biological response modifiers, J. Control Release 130 (2) (2008) 98–106,]. Iron concentration in the final NPs solutions, determined by ferrozine assay, were 5.0 and 4.8 mg/ml for Magn and Magn-Plu samples, respectively. The value of pH was close to a neutral one.

Size and morphology of obtained samples were investigated by TEM method. In both cases the shape of NPs was close to a spherical one; the average diameter of Magn NPs was slightly smaller than that of Magn-Plu NPs (the values of 9.0±2 nm and 11.1±2 nm were obtained, respectively). TEM micrographs of the samples and corresponding size distribution of NPs are displayed in Fig. S3.

| 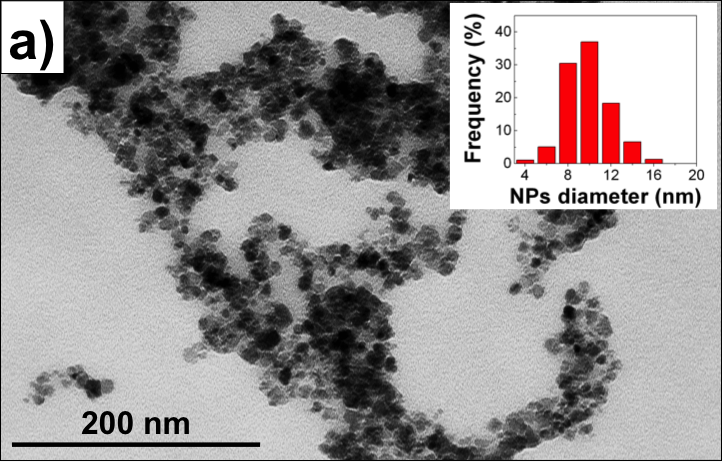 | 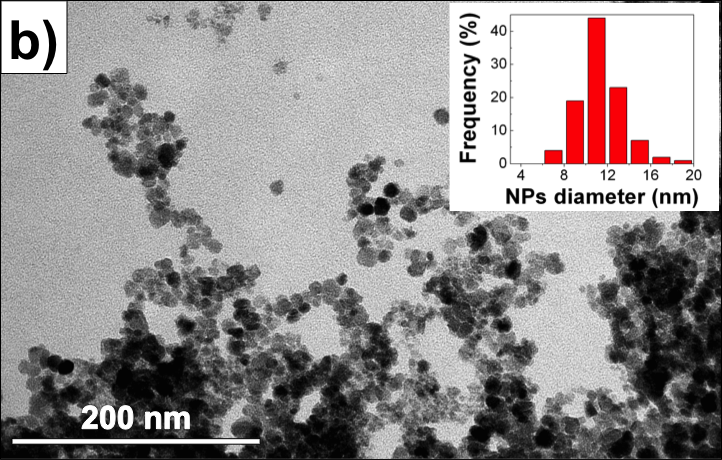 |
| --- | --- |

**Fig. S3** TEM-micrographs of Fe_3_O_4_ NPs: a) without coating (sample Magn) and b) with Pluronic F127 coating (sample Magn-Plu). The inserts display the size distribution of NPs

Phase composition of the samples was confirmed by the results of XRD-analysis (see Fig. S4) – the position and relative intensity of all peaks correspond to Fe_3_O_4_ structure.


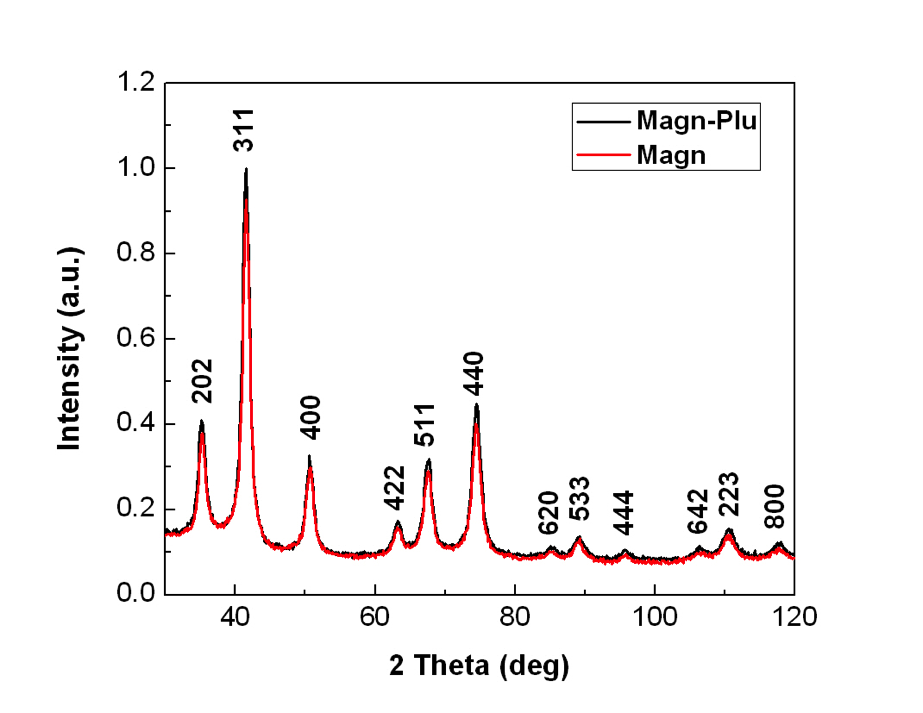


**Fig. S4.** X-Ray diffraction patterns of Magn and Magn-Plu NPs. The indexes correspond to Fe_3_O_4_ phase. The intensity is normalized to (311) peak*.*

The hydrodynamic radius and also zeta-potential of NPs, measured by DLS method, are the important parameters regulating the stability of NPs. Fig. S5 displays the distribution of hydrodynamic radii of Magn and Magn-Plu NPs.


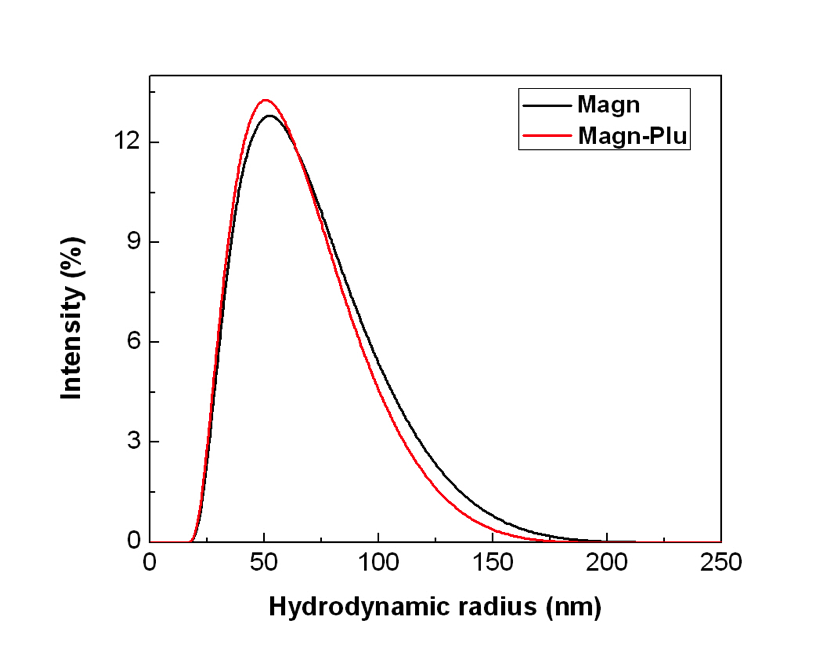


**Fig. S5***.* Size distribution of Magn and Magn-Plu NPs measured by DLS method

One can see that some difference in size measured by TEM method doesn’t affect the effective radius of NPs in the solution. The average values of size/zeta-potential are 104±22 nm/ -32±5 mV for Magn sample and 100±18 nm/ -13±4 mV for Magn-Plu sample, respectively. Negative zeta-potential of the samples is caused by the presence of OH^-^- ions on the surface of Magn NPs and negative-charged polymeric chains of Pluronic F127 on the surface of Magn-Plu NPs.

*Stability of* *catalytic activity of platinized electrode during intracellular measurements*

*
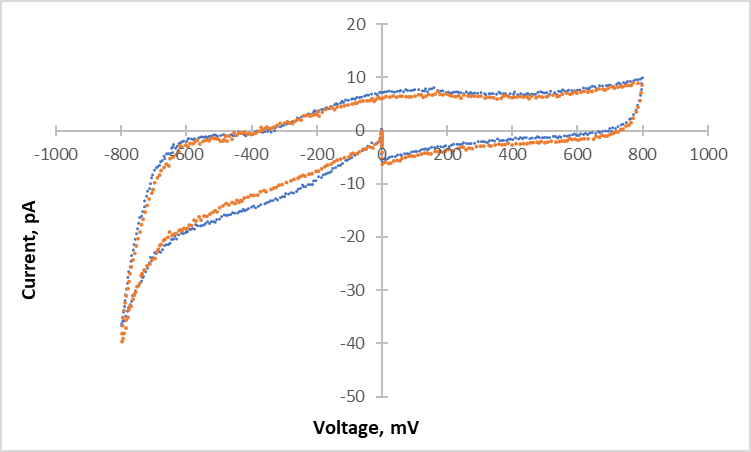
*

**Fig. S6** Catalytic activity for oxygen reduction of nanoelectrodes vs Ag/AgCl. Cyclic voltammograms in HBSS buffer solution: yellow graph –initial platinized electrode; blue graph – platinized electrode after intracellular measurements.

*Apoptosis/necrosis detection*


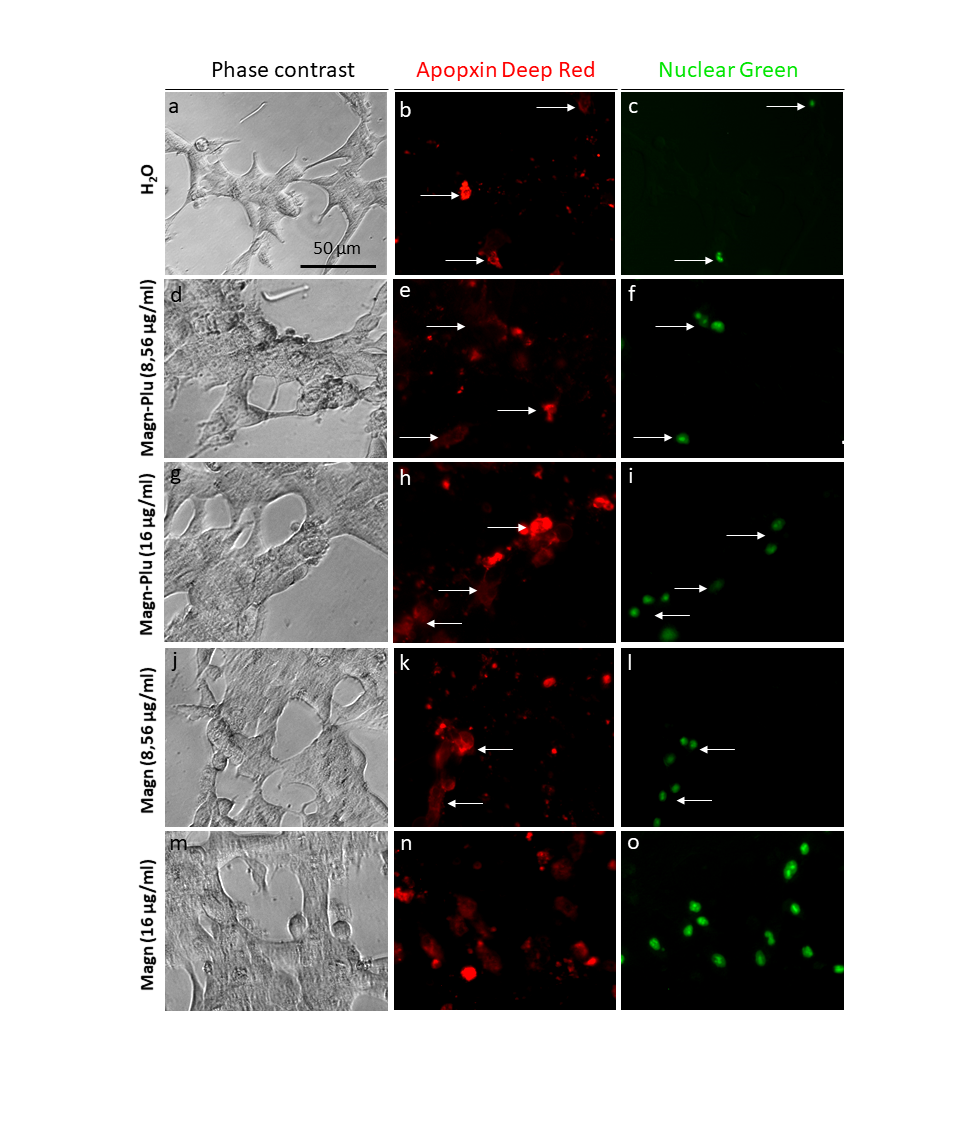


**Fig. S7** Detection of dead cells in cell population after incubation with Magn-Plu and Magn nanoparticles during 24 h. Intravital staining with apoptosis/necrosis kit, fluorescent microscopy. Arrows show dead cells.
